# Evaluation of the effect of stent strut profile on shear stress distribution using statistical moments

**DOI:** 10.1186/1475-925X-8-8

**Published:** 2009-04-30

**Authors:** Juan Mejia, Bilal Ruzzeh, Rosaire Mongrain, Richard Leask, Olivier F Bertrand

**Affiliations:** 1 Department of Mechanical Engineering, McGill University, Montreal, Quebec, Canada; 2 Department of Chemical Engineering, McGill University, Montreal, Quebec, Canada; 3 Quebec Heart-Lung Institute, Laval Hospital, Quebec City, Quebec, Canada; 4 Montreal Heart Institute, Montreal, Quebec, Canada

## Abstract

**Background:**

In-stent restenosis rates have been closely linked to the wall shear stress distribution within a stented arterial segment, which in turn is a function of stent design. Unfortunately, evaluation of hemodynamic performance can only be evaluated with long term clinical trials. In this work we introduce a set of metrics, based on statistical moments, that can be used to evaluate the hemodynamic performance of a stent in a standardized way. They are presented in the context of a 2D flow study, which analyzes the impact of different strut profiles on the wall shear stress distribution for stented coronary arteries.

**Results:**

It was shown that the proposed metrics have the ability to evaluate hemodynamic performance quantitatively and compare it to a common standard. In the context of the simulations presented here, they show that stent's strut profile significantly affect the shear stress distribution along the arterial wall. They also demonstrates that more streamlined profiles exhibit better hemodynamic performance than the standard square and circular profiles. The proposed metrics can be used to compare results from different research groups, and provide an improved method of quantifying hemodynamic performance in comparison to traditional techniques.

**Conclusion:**

The strut shape found in the latest generations of stents are commonly dictated by manufacturing limitations. This research shows, however, that strut design can play a fundamental role in the improvement of the hemodynamic performance of stents. Present results show that up to 96% of the area between struts is exposed to wall shear stress levels above the critical value for the onset of restenosis when a tear-drop strut profile is used, while the analogous value for a square profile is 19.4%. The conclusions drawn from the non-dimensional metrics introduced in this work show good agreement with an ordinary analysis of the wall shear stress distribution based on the overall area exposed to critically low wall shear stress levels. The proposed metrics are able to predict, as expected, that more streamlined profiles perform better hemodynamically. These metrics integrate the entire morphology of the shear stress distribution and as a result are more robust than the traditional approach, which only compares the relative value of the local wall shear stress with a critical value of 0.5 Pa. In the future, these metrics could be employed to compare, in a standardized way, the hemodynamic performance of different stent designs.

## Introduction

Recently stent design was directly linked to in-stent restenosis rates [1]. For example, the corrugated ring stent design was found to result in smaller tissue proliferation than tubular slotted stent design suggesting that vessel response is dependent on stent design [2-6]. Since then a great deal of effort has been invested to improve stent designs. In general, the overall aim has been set toward improving the hemodynamic compatibility of stents.

A factor that has proved to be a strong predictor of in-stent restenosis is the stent's strut thickness, where thicker struts result in higher restenosis rates when compared to thinner strut designs [7]. Other studies have found a link between stent and neointimal thickening observed in human and animal experiments after vascular remodeling occurred [8,9].

Work on the effect of wall shear stress (WSS) on the arterial wall have been able to find a strong relationship between abnormal regions of WSS with the generation of mitogens that can lean to neointimal hyperplasia (NIH), which can cause in-stent restenosis [10-12]. It has also been shown that the stent's presence can result in recirculation and reattachment regions between individual struts, and that the characteristics of these flow stagnation regions are dependent on strut spacing and geometry [13-22]. Moore *et al*. suggested that zones of recirculation and stagnant blood flow created by stenting are precursors of restenosis [23].

Moreover, it has been suggested that very high shear stress created along the stent's struts is a factor that could potentially cause in-stent restenosis [24] due to the alteration of blood constituents [25]. All these observations suggest that improvements of stent design could potentially lead to a decrease in restenosis rates.

In this paper, we study the impact of strut cross-sectional profile on the wall shear stress distribution along a stented segment of a coronary artery. Four different strut cross-sectional profiles (square, circular, elliptical, and tear-drop) are investigated. Simple metrics are suggested to assess the deviation of the shear stress distribution along the wall from the reference condition – i.e. an unstented arterial segment. It is shown that the proposed metrics are coherent with the fact that more streamlined profiles perform better than the more blunt profiles hemodynamically.

## Methods

### Mathematical Model

To illustrate the approach the geometry of a stented arterial segment is idealized. The three-dimensional arterial geometry is assumed to be axis-symmetric and therefore a 2D representation is used. In the discussion section methods of application to 3D stent geometries are presented. The blood is modeled as a Newtonian fluid. The flow is studied under laminar steady state conditions. A fully developed velocity profile is imposed at the entrance of the vessel, and the Reynolds number corresponds to the average condition over one cycle [16]. The governing equations are then the following simplified Navier-Stokes equations:

(1)

(2)

where *ρ *is the blood density (kg/m^3^), *μ *is the blood dynamic viscosity (Pa·s), p is the pressure (Pa), and  is the velocity field of the blood (m/s).

### Numerical Model

**Figure 1:**
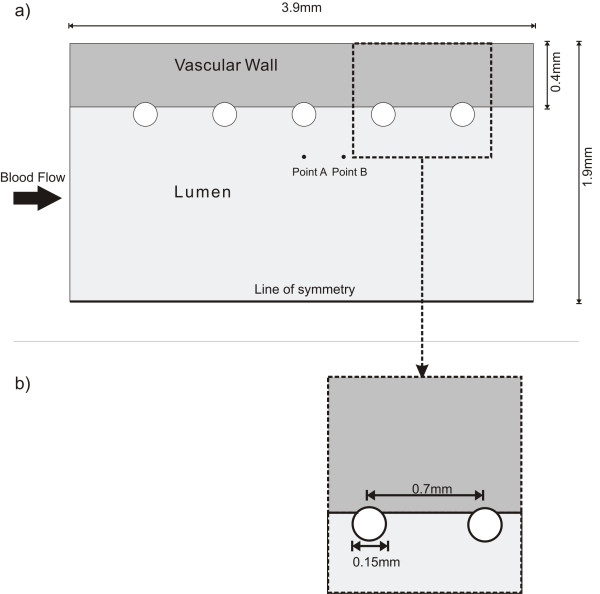
**Geometrical model**. a) Overall Longitudinal Section View of the stented arterial segment; b) detailed view of strut geometry.

The numerical mesh of the stent and vessel are created using GAMBIT^© ^(FLUENT Inc). Figure 1a shows a representation of a stented artery segment with five successive non-embedded struts. Similar stent configurations have been used in previous studies [26,27].

**Figure 2:**
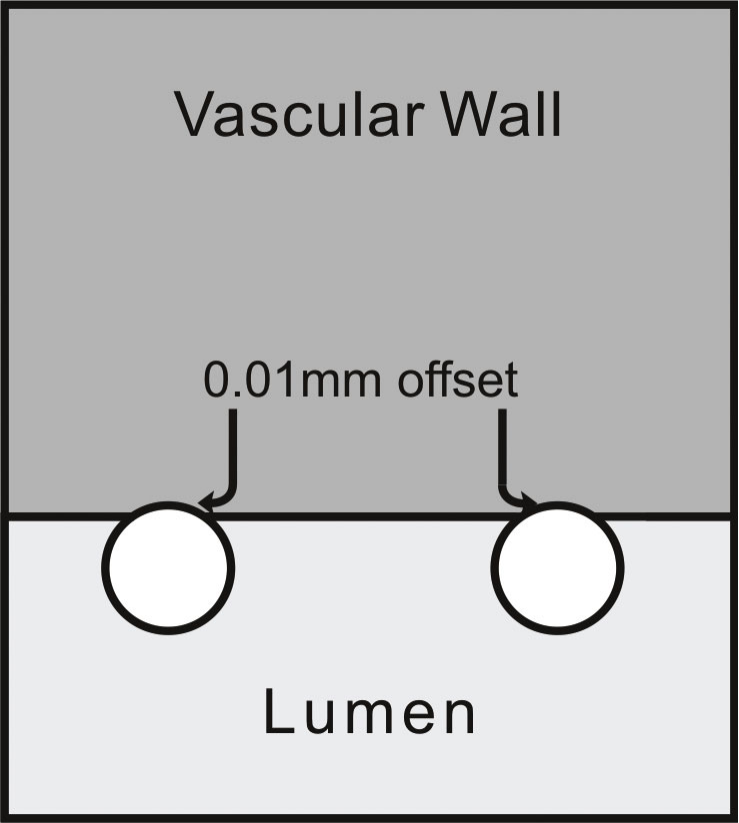
**Close-up of geometrical model**. Strut apposition configuration.

The relevant dimensions used in this configuration are adopted from typical anatomical values of coronary artery dimensions. A 3.0 mm inner diameter and 0.4 mm wall thickness are selected [28]. The generic stent dimensions are: strut diameter of 0.15 mm and inter-strut distance (ISS) of 0.7 mm (from center to center), with a total vessel length of 3.9 mm (Figure 1). These stent dimensions are typical of common coronary stents [29] and the wall thickness corresponds to a moderately thickened wall since a normal coronary intima-media thickness is about 200 *μ*m [30]. Four different strut cross-sectional profiles -square, circular, elliptic, and tear drop- were studied; keeping the initial strut thickness (0.15 mm) constant, and implementing a 0.01 mm offset toward the interface (Figure 2). This offset is used to avoid the singularity problem that arises numerically with a single contact point. The analysis is performed for the non-embedded and half-embedded configuration (simply apposed on the vascular wall).

**Figure 3:**
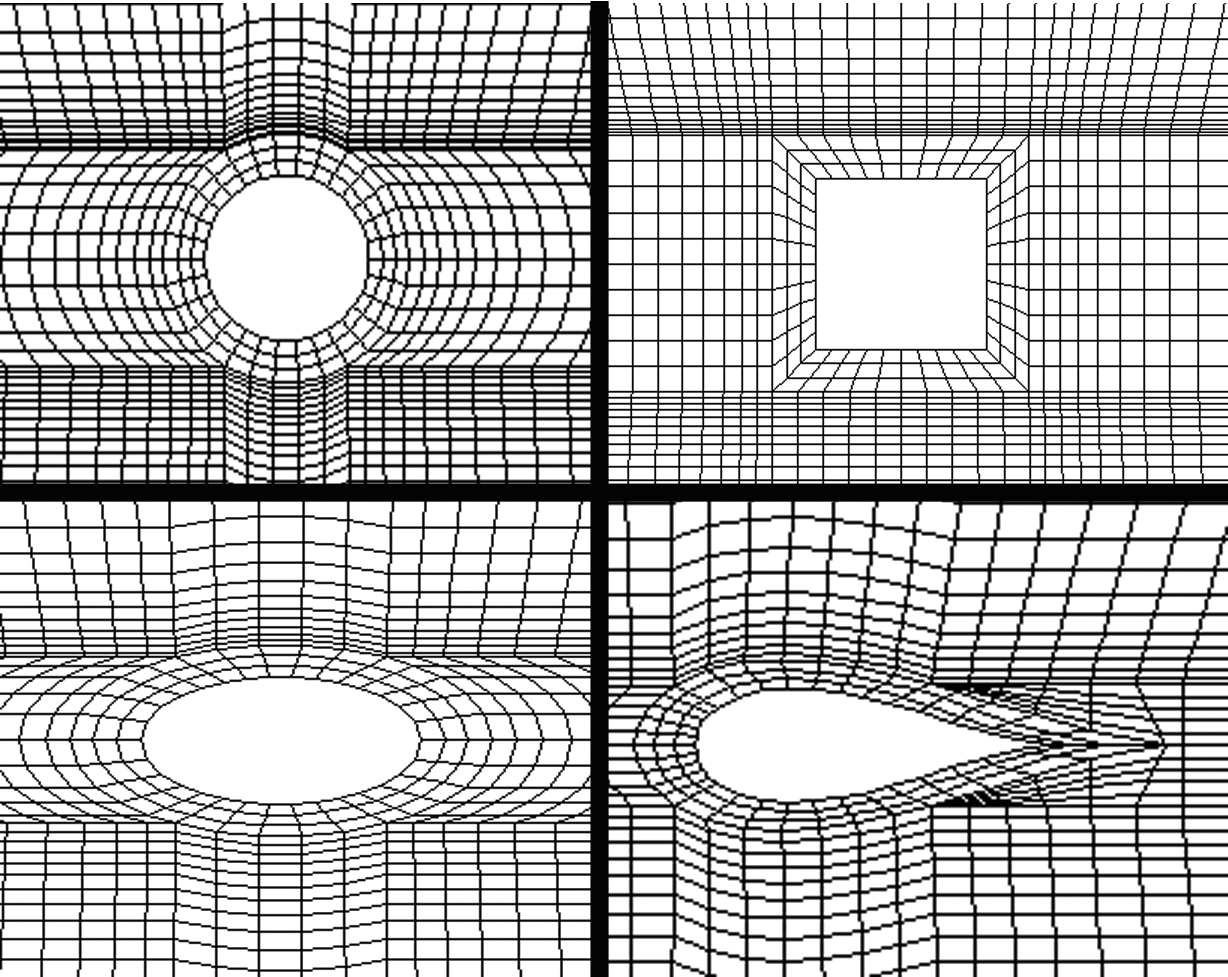
**Finite element model**. Mesh pattern around the different strut profiles. Counterclockwise from top-left: circular, square, elliptical, and tear-drop cross-section.

Different mesh schemes were tested and a mesh dependency test was run using FIDAP^© ^(Fluent Inc). Accordingly, the convergence was analyzed at two points, labeled points A and B (Figure 1), around the third strut in terms of the x-component of the velocity and the shear stress. The location of point A is 250 *μ*m away for the vascular wall and centered to the strut face and point B is at the same distance from the vascular wall and located halfway between the third and fourth struts. Values obtained at A and B show that the selected linear quadrilateral elements exhibit mesh independence with 14550 elements. The resulting meshes for the different strut profiles are illustrated in Figure 3.

A working fluid with a density of 1057 kg/m^3 ^and a dynamic viscosity of 3.5 mPa.s was used. At the inlet, a parabolic velocity profile is imposed (corresponding to a Reynolds number of 240 [31]), and zero velocity components in the radial and circumferential directions are assumed. The resulting average flowing speed is 0.265 m/s, which corresponds to physiological values. The vessel wall is assumed to be rigid in this study. The radial and circumferential velocity components are assumed to be equal to 0 at the inlet, with a no-slip boundary condition at the walls. A more detailed description of the boundary conditions used in the numerical simulations can be found in Table 1. The results for each strut profile are then compared on the basis of their shear stress distribution variation with respect to the reference non-stented case.

**Table 1:** Summary of boundary conditions.

Boundary Conditions	
Computational Domain	*V*_*θ *_= 0

Inlet	*V*_*r *_= 0
	*V*_*z *_= 0.265(1 - (*r*/*R*)^2^) (parabolic velocity profile)

Symmetry Line	*V*_*r *_= 0

Wall-blood interface	*V*_*z *_= 0 (no-slip condition)
	*V*_*r *_= 0 (non-porous wall)

Summary of boundary conditions used for the numerical simulations.

### Shear Stress Metrics

The shear stress is evaluated on all solid walls (stent and vessel walls) using FIDAP^© ^built-in routines for the four cross sectional profiles. In FIDAP, for a Newtonian fluid, the shear rate tensor (*τ*) is related to the shear stress tensor by the following linear relation:

(3)

where *μ *is the dynamic viscosity and *s*_*ij *_the shear rate tensor. Therefore, for a Newtonian fluid, the shear stress and shear rate plots exhibit the same general morphology.

Quantitatively, the global WSS distributions can be compared using statistical moments such as the mean, standard deviation, and kurtosis. We have limited our present analysis to the region between the first and second strut, while assuming that results obtained within this region remain for the entire stented arterial segment.

Briefly, the nth central moment of the function *τ*(x) about a constant, c, is:

(4)

where x is the spatial dimension along which *τ *is evaluated. With this definition, the first central moment about zero is the mean of the function. the second central moment about the mean is the variance, the square root of which is the standard deviation. The third central moment is a measure of the lopsidedness of the distribution; a perfectly symmetric distribution will have a third central moment of zero. The third central moment is not employed here as it is assumed that the overall shift of the WSS is not as important as its flatness and elevation, which are measured by the first, second, and third statistical moments. Finally, the fourth central moment, also referred to as the kurtosis, is a measure of whether the distribution is tall and skinny (leptokurtosis), or short and squat (platokurtosis). The kurtosis is defined as the standardized fourth central moment. For a flat distribution (Poiseuille shear stress distribution), the average value at the wall is constant, the standard deviation is zero, and the skewness and kurtosis are zero. In fact, this approach is borrowed from tribology where similar metrics are employed to characterize surface roughness [32].

The first and second statistical moments, the mean and standard deviation respectively, have units of Pascals. We have non-dimensionalized them by dividing the first statistical moment by the average WSS of a normal artery (2.5 Pa as calculated with a Poiseuille flow) and by dividing the second statistical moment by the first – also known as the coefficient of variation.

The fourth statistical moment – the kurtosis – is by definition non-dimensional, and we have related the kurtosis to the mean by multiplying it with the non-dimensionalized mean, and calling it the Kurtosis Coefficient. Mathematically this can be expressed as:

(5)

For the case of a non-stented artery the last term of (5) is 1, in which case the kurtosis and the kurtosis coefficient are identical. When the mean changes – due to the presence of the stent – the kurtosis is greater than the kurtosis coefficient. Therefore, (5) is a measure of the distribution's flatness as well as its overall elevation.

## Results

**Figure 4:**
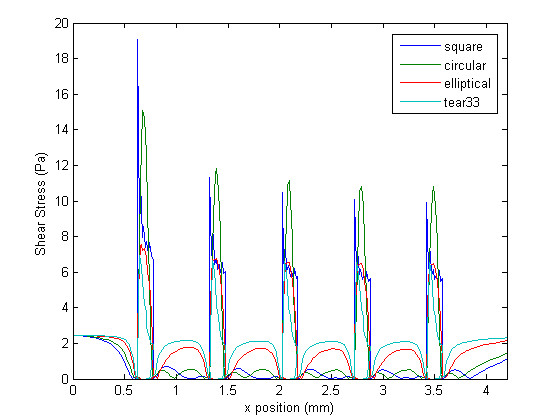
**Wall shear stress plot**. Shear stress at the wall for different strut profiles in the non-embedded scenario.

**Figure 5:**
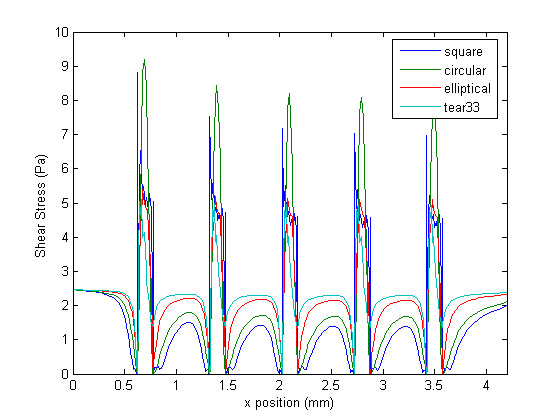
**Wall shear stress plot**. Shear stress at the wall for different strut profiles in the half-embedded scenario.

Figure 4 shows the wall shear stress (WSS) generated along the arterial wall for the different non-embedded strut profiles under study. In this figure, as well as in all other figures, blood flows from left to right. In general, a WSS of approximately 2.5 Pa is observed at the entrance of the channel – before the beginning of the stented region. The first strut, located at approximately 0.6 mm downstream of the entrance, causes a spike in WSS, which is immediately followed by a region of relatively low WSS. Levels are lowest in the region adjacent to the struts. They then increase with distance from the struts, and are, in general, highest in the region furthest away from the struts (i.e. approximately half-way between two consecutive struts), with the exception of the WSS on the struts themselves. In other words, WSS spikes are observed on the struts and WSS valleys are observed between the struts for all strut profiles. The shear stress peaks gradually decrease as the flow passes over the consecutive struts. Figure 5 shows the WSS distribution obtained with the half-embedded struts profiles. On average, WSS levels between struts are higher from those obtained with the non-embedded struts. The maximum value of WSS never exceeded the WSS value observed along the non-stented wall for either the non-embedded or half-embedded struts. For the half-embedded struts, however, the WSS increases faster with distance from the struts. In fact, for the case of the non-embedded struts the circular and square profiles exhibit very low levels of WSS along the entire distance between two consecutive struts. For the case of the half-embedded struts the square and circular profile exhibit a much more favorable WSS distribution in comparison to the non-embedded case, albeit not as favorable as those obtained with the elliptical and tear-drop profiles. The WSS spikes located on the struts are significantly higher for the non-embedded struts, with the largest WSS value being twice as high as the highest WSS value obtained with the non-embedded struts.

**Figure 6:**
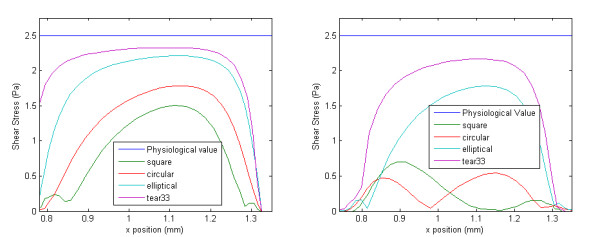
**Partial wall shear stress plot**. Wall shear stress – between the first and second strut – for different strut profiles. Right: strut is non-embedded, and Left: strut is half-embedded

Qualitatively it is possible to compare the WSS distributions of each strut profile. The square and circular profiles have the highest WSS peaks, while the tear-drop and elliptical profiles have the lowest peaks. Furthermore, the WSS between struts are significantly higher for the elliptical and tear-drop profiles, while the square and circular profiles show the lowest levels of WSS. These results suggest, as expected, that more streamlined strut profiles causes less flow disturbances, which confirms that the proposed metrics are coherent. For the case of the half-embedded struts (Figure 5), representing a stented arterial segment several weeks after implantation, the general flow behavior remains similar, but WSS spike levels are lower by approximately 40%, and WSS levels between struts are, on average, approximately twice as high. Figure 6 shows the WSS levels between the first and second struts for both the half-embedded and non-embedded struts. The atheroprotective WSS, corresponding to the physiological level found in an unstented vessel, is also plotted as a reference value. As expected, the WSS distribution obtained with the tear-drop and elliptical profiles are higher in both the non-embedded and half-embedded scenarios. The WSS distribution for both the circular and square profiles are low, although they significantly improve for the half-embedded case.

The statistical moments of the WSS shown in Figure 6 were calculated for both the non-embedded and half-embedded cases (Tables 2 and 3 respectively). In general, the WSS distribution of the more streamlined strut profiles have a higher non-dimensional mean, lower coefficient of variation, and higher kurtosis coefficient.

**Table 2:** Statistical moments.

Non-embedded struts			
Profile	Non-dimensional Mean	Coefficient of Variation	Kurtosis Coefficient

Square	0.0936	1.0212	0.2099

Circle	0.0865	0.8908	0.1355

Ellipse	0.3034	0.9404	0.4176

Tear Drop (1:3)	0.4732	0.7715	0.6091

Statistical moment for the case of the non-embedded struts.

**Table 3:** Statistical moments.

Half-embedded struts			
Profile	Non-dimensional Mean	Coefficient of Variation	Kurtosis Coefficient

Square	0.2618	0.8395	0.3916

Circle	0.3851	0.6817	0.5797

Ellipse	0.6259	0.4496	1.6703

Tear Drop (1:3)	0.7679	0.3127	4.7543

Statistical moment for the case of the half-embedded struts.

## Discussion

Present results show that the proposed metrics correctly assess the hemodynamic performance of different strut profiles. Indeed they confirm that the tear-drop and elliptical strut profile perform best in both the non-embedded and half-embedded scenarios. The results revealed that the stent strut profile has a significant impact on the wall shear stresses both on the struts and in between struts. It also showed that slender and streamlined profiles provide better results in terms of peak stress. Tear-drop and elliptical profiles have better performance than the classical square and circular profiles. Furthermore, the current work suggests that appropriate strut apposition can lead to a significant improvement in terms of the hemodynamic performance of a stent.

The authors feel there is a need for appropriate data reduction analysis, and standardization, in the field of stent design, because of the complexity of the numerical studies and the various ways of assessing the hemodynamic performance available in the literature, which render comparison amongst studies difficult. Such considerations provided the motivation for this study, resulting in a set of metrics that have the capability of assessing the global WSS morphology and comparing it to a common standard.

Results showed that the metrics proposed here are in agreement with the traditional qualitative ways of evaluating hemodynamic performance. In almost all cases, the non-dimensional mean increases as the strut profile becomes more streamlined. The only exception is observed when the strut profile changes from a square to a circle in the non-embedded case. This exception can be explained by the fact that a circular profile comes into contact with the arterial wall further upstream than the square profile as the distance from the center of the strut is always constant for the circular geometry. Assuming that restenosis rates are reduced when the flow within the stented region is as similar to physiological values as possible the ideal stent would have a non-dimensional mean of 1. As such an ideal stent does not exist, it is assumed that the higher the non-dimensional mean the less likely it is that restenosis will occur. A similar argument is true for the kurtosis coefficient. A high kurtosis coefficient implies that the distribution is both relatively flat and contains, on average, high values of WSS. Therefore, a stent design with high kurtosis coefficient is desirable. It is not sufficient, however, to take into account the non-dimensional mean without the kurtosis coefficient as the first statistical moment is sensitive to extreme isolated values. Finally, the coefficient of variation of WSS within the stented region for a normal unstented artery is naturally low, which implies that a stent design with low coefficient of variation is desirable. Results from Tables 2 and 3 support this argument as the WSS distributions obtained with the elliptical and tear-drop profiles have the lowest coefficient of variation.

A stent design with a low coefficient of variation, high coefficient of kurtosis, and high non-dimensional mean is superior to a stent design with high coefficient of variation, high coefficient of kurtosis, and high non-dimensional mean as the latter probably has a high outlying value which is influencing the results, while it is possible that the overall WSS is relatively low. In general, when comparing stent designs in terms of their resulting WSS distributions a stent with high non-dimensional mean, high coefficient of kurtosis, and low coefficient of variation is assumed desirable. In the past, several studies have described the overall morphology of the WSS within a stented region [26,27,33] but as of yet a quantitative method of evaluating the global WSS distribution has not been presented.

As a way of verifying our conclusions the percent of the vessel area per unit depth, between the first and second struts, that is exposed to a WSS higher than 0.5 Pa was calculated. This procedure was carried out for each strut profile in both the non-embedded and half-embedded scenarios. A similar approach was used to evaluate the hemodynamic performance of different stent designs by Balossino *et al*. [34]. The critical value of 0.5 Pa has been reported in previous work as a threshold for the onset of in-stent restenosis [35]. The results are presented in Tables 4 and 5. In general, the more streamlined strut profiles have a higher percentage of inter-strut area where the WSS level is over the critical value. Calculating the percent area exposed to critically low WSS levels does not, however, integrate the global morphology of the WSS into the assessment of hemodynamic performance. The proposed metrics, on the other hand, provide a standardized way of integrating the global shear stress morphology into the evaluation of the stent's hemodynamic performance. They have the capacity to measure the hemodynamic performance even if two different stent designs result with the same percentage of the stented area over the critical WSS value. They also measure the variation, flatness, and overall elevation of the WSS distribution. Therefore, evaluating the proposed metrics is a more robust way of determining hemodynamic performance.

**Table 4:** Percentage of area over critical value.

Non-embedded struts	
Profile	% of area over threshold value

Square	19.4

Circle	9.7

Ellipse	50

Tear Drop (1:3)	61.3

Percent of area over critical value for the case of the non-embedded struts.

**Table 5:** Percentage of area over critical value.

Half-embedded struts	
Profile	% of area over threshold value

Square	45

Circle	67.7

Ellipse	90.3

Tear Drop (1:3)	96.2

Percent of area over critical value for the case of the half-embedded struts.

The principal objective of this study was to develop the general methodology and simple metrics to assess the shear stress distribution associated with various stent strut designs. The present work shows that the proposed metrics are effective as they confirm that more streamlined cross-sectional strut profiles have better hemodynamic performance. Presently, struts with square cross-sectional profiles are common, but current results show that this type of profile might hamper hemodynamic performance. Elliptical profiles, which perform better according to the metrics proposed here, can be manufactured by chemically etching rectangular profiles obtained from conventional laser cut tubes. More complex profiles, such as the tear drop profile, would require more elaborate manufacturing processes. The tear drop profile was used because of its well known fluid dynamic properties. These properties provide a way to assess the proposed metrics for comparison purposes. In fact, the conclusions drawn from this study would not change if a tear-drop profile with a blunt trailing edge would have been used. Indeed, the circular, elliptical, and tear drop profiles have been investigated in the past in terms of their shear stress distribution [36]. In that reference it was shown that elliptical profiles have very close properties to the tear drop profile.

Valuable insight has been obtained by analyzing the present two-dimensional model. Although future work should also include more complicated model of the stented vessel (e.g. including curvature and compliance) there are still important lessons that can be learned from relatively simple simulations, as was also recently demonstrated by Kolachalama *et al*. and Borghi *et al*. [37,38].

**Figure 7:**
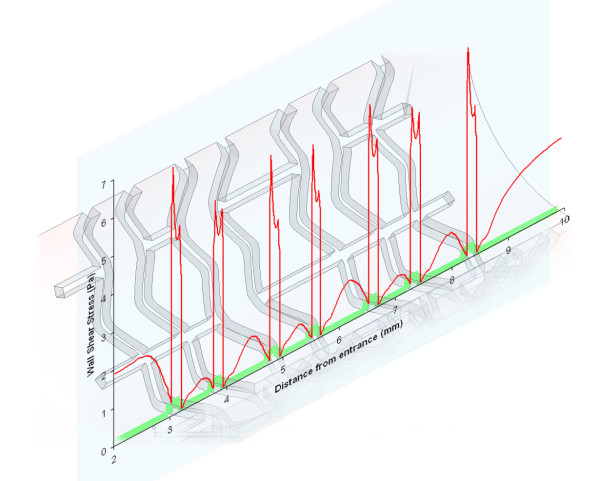
**Wall shear stress plot of a 3D transient model**. WSS distribution obtained by Mejia *et al*. using a fully 3D transient model of a relaistic stent geometry.

**Figure 8:**
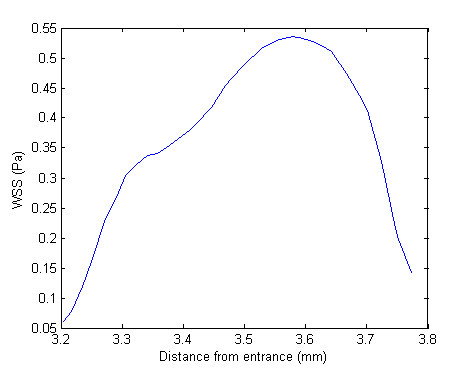
**Partial wall shear stress plot**. Close-up of WSS distribution corresponding to the region between the first and second struts of Figure 7.

The authors have previously produced numerical simulations using three-dimensional transient models to which the proposed metrics can be applied [39,40]. Figures 7 and 8 show the WSS along a selected two-dimensional section and the corresponding WSS distribution between the first two struts, respectively. These profiles were obtained along an arbitrary line created by the intersection of the arterial model and a plane oriented in the axial direction. In fact, LaDisa *et al*. recently presented a study in which two-dimensional sections, such as those already discussed here, were used to evaluate relative hemodynamic performance – in terms of the overall WSS magnitude [22]. The values correspond to a fully three-dimensional transient model of a realistic stent geometry [41,42] with square strut profiles (instantaneous Reynolds number of 190, and 0.3 seconds after the start of diastole) [40]. The resulting metrics for this WSS distribution are 0.31, 0.40, and 0.64 for the non-dimensional mean, coefficient of variation, and kurtosis coefficient, respectively. In comparison to the results obtained with the two-dimensional models presented here these results are off by approximately a factor of 3. Both the coefficient of kurtosis and the non-dimensional mean are approximately 3 times higher while the coefficient of variation is approximately 3 times lower, when calculated with the three-dimensional results. In conclusion the three-dimensional transient model predicts more favorable WSS distribution, which can be accounted for by the three-dimensionality of the flow. This implies that the use of a two-dimensional model can potentially lead to an overestimation of the restenosis risk. The proposed metrics can be readily applied to two-dimensional sections of a three-dimensional models, and can even be used to quantitatively compare results obtained by different research groups against a common standard, as was just demonstrated. Another more general way of applying the proposed metrics to a three-dimensional model would be to replace the line integral of (4) by an appropriate surface integral [43]. For example, instead of calculating the moments of inertia along the line created by the vessel surface of a three-dimensional model and a plane oriented in the axial direction – as in equation 4 – they can be calculated over the surface of the vessel enclosed by one stent cell. Mathematically this can be expressed as:

(6)

Consequently, the proposed metrics can be calculated on a two-dimensional section of a three-dimensional model, or over a three-dimensional surface.

This work assumed that blood can be modeled as a Newtonian fluid. However, recent studies have suggested that the non-Newtonian nature of blood can have a non-negligible impact on WSS levels [44]. Therefore, future work should include the non-Newtonian behavior of blood into the model. The present work also assumed rigid vessel walls, which could potentially alter WSS distributions and should also be investigated in the future.

## Conclusion

In this work, we presented simple metrics to assess and compare the shear stress levels associated with various stent strut profiles. The metrics are defined with respect to the reference values of the corresponding normal unstented arterial segment. In other words, we globally assess the difference of the shear stress distribution between the stented and the unstented conditions. Although there are several methods presented in the literature to asses stent performance – such as those used by Moore *et al*. and Balossino *et al*. [17,34] – the proposed metrics introduce the first standardized method of assessing hemodynamic performance in terms of WSS distribution.

As expected, results suggest that more streamlined strut profiles exhibit better hemodynamic performance.

In addition, when comparing the non-embedded and the half-embedded scenarios the latter exhibits more favorable WSS distributions for the same strut profile, which is also to be expected. In terms of the proposed metrics, both the streamlined profiles and the half-embedded struts perform better. Therefore, it is concluded that the metrics introduced in this work can be later used to assess the impact of more complex factors – such as stent cell geometry, inter-strut distance, strut thickness, etc. – on WSS distribution. Although these metrics were proposed here in the context of a 2D model they can be directly applied to any WSS distribution regardless of how it is obtained (i.e. 3D numerical model, *in-vitro *model, or *in-vivo*). Future work should include dedicated clinical trials to provide a direct link between the proposed metrics and restenosis rates.

## Competing interests

The authors declare that they have no competing interests.

## Authors' contributions

BR carried out the 2D simulation and helped drafting the initial manuscript. RM and RL conceived the project, supervised it, analyzed the final results, and helped drafting the manuscript. JM carried out additional computations, manipulated the raw data, analyzed the results, revised the manuscript, and drafted the final version. OB provided the clinical context and valuable medical advice, as well as revising the final draft.
